# Conditional relative survival in primary central nervous system lymphoma: a population-based study in the Netherlands

**DOI:** 10.1093/noajnl/vdaa133

**Published:** 2020-10-01

**Authors:** Avinash G Dinmohamed, Matthijs van der Meulen, Otto Visser, Jeanette K Doorduijn, Jacoline E C Bromberg

**Affiliations:** 1 Department of Research and Development, Netherlands Comprehensive Cancer Organisation (IKNL), Utrecht, The Netherlands; 2 Erasmus MC, University Medical Center Rotterdam, Department of Public Health, Rotterdam, The Netherlands; 3 Erasmus MC Cancer Institute, University Medical Center Rotterdam, Department of Neuro-oncology, Rotterdam, The Netherlands; 4 Department of Registration, Netherlands Comprehensive Cancer Organisation (IKNL), Utrecht, The Netherlands; 5 Erasmus MC Cancer Institute, University Medical Center Rotterdam, Department of Hematology, Rotterdam, The Netherlands

**Studies on conditional relative survival in primary central nervous system lymphoma (PCNSL) have hitherto been lacking in the literature. Using data from the Netherlands Cancer Registry, we examined the conditional 5-year relative survival up to 5-year postdiagnosis among PCNSL patients in the Netherlands. An encouraging finding of our study was that excess mortality decreased after each additional year survived postdiagnosis. However, on the other side of the pendulum, conditional 5-year relative survival did not exceed 95%. This finding indicates that PCNSL patients continue to experience substantial excess mortality, as compared to age- and sex-matched groups from the general population.**

PCNSL is a rare and highly aggressive non-Hodgkin lymphoma restricted to the brain, leptomeninges, spinal cord, and eyes. The population-level survival of PCNSL patients improved significantly over the past decades—mostly due to the increased use of high-dose methotrexate-based chemotherapy—albeit confined to patients up to age 70.^1,2^ Despite the encouraging progress, the unfortunate reality remains that a high relapse rate characterizes PCNSL and much of the mortality occurs within the first few years postdiagnosis.^1–4^ In considering the latter, it is of interest to establish the prognosis after having survived a specified time since diagnosis (ie, conditional survival). Statistics on conditional survival that have been corrected for the life expectancy in the general population are lacking in PCNSL (ie, conditional relative survival). Therefore, this nationwide, population-based study aimed to examine up-to-date conditional 5-year relative survival estimates among PCNSL patients in the Netherlands.

We selected all patients diagnosed with PCNSL of the diffuse large B-cell type between 1989 and 2018—with survival follow-up through December 31, 2019—from the Netherlands Cancer Registry, which ascertains all newly diagnosed malignancies in the Netherlands since 1989 through multiple notification sources. Details about the registry are described elsewhere.^1^ PCNSL was confirmed by histology, cytology, and/or flow cytometry and defined using International Classification of Diseases for Oncology morphology and topography codes as described elsewhere.^1^ The Privacy Review Board of the Netherlands Cancer Registry approved the use of anonymous data for this study.

We calculated 5-year relative survival at diagnosis and the conditional 5-year relative survival for each additional year survived up to 5 years postdiagnosis, conditional on being alive at the beginning of that year. Survival estimates were presented for the overall cohort and stratified by sex and age at diagnosis (18–50, 51–60, and >60 years). Relative survival was calculated to estimate disease-specific survival as the ratio of the patients’ overall survival to the expected survival, based on national population life tables, stratified by age, sex, and calendar year.^5^ Excess mortality, as compared to the general population, is considered minimal when 5-year relative survival exceeds 95%.^6^ Differences in survival estimates between subgroups were considered statistically significant when the 95% CIs do not overlap.

The hybrid approach—which was described and empirically validated by Brenner and Rachet and is conceptually similar to the approach used to estimate the life expectancy at birth—was used to compute up-to-date survival estimates in the setting where incidence data lag behind mortality statistics by left-truncating the survival probabilities at the start of the period of interest, besides right-censoring it at its end.^7^ It was applied for patients diagnosed during 1989–2018 who were alive during the follow-up interval 1998–2019. The estimates produced by the hybrid approach herein can be interpreted as the predicted survival probabilities for patients diagnosed during 1998–2019. The manner how the survival data was constructed under the hybrid approach resulted in 10 years of postdiagnostic follow-up information to compute the conditional 5-year relative up to 5 years postdiagnosis. Brief details about the statistical analyses are described in the figure legend.

**Figure 1.**
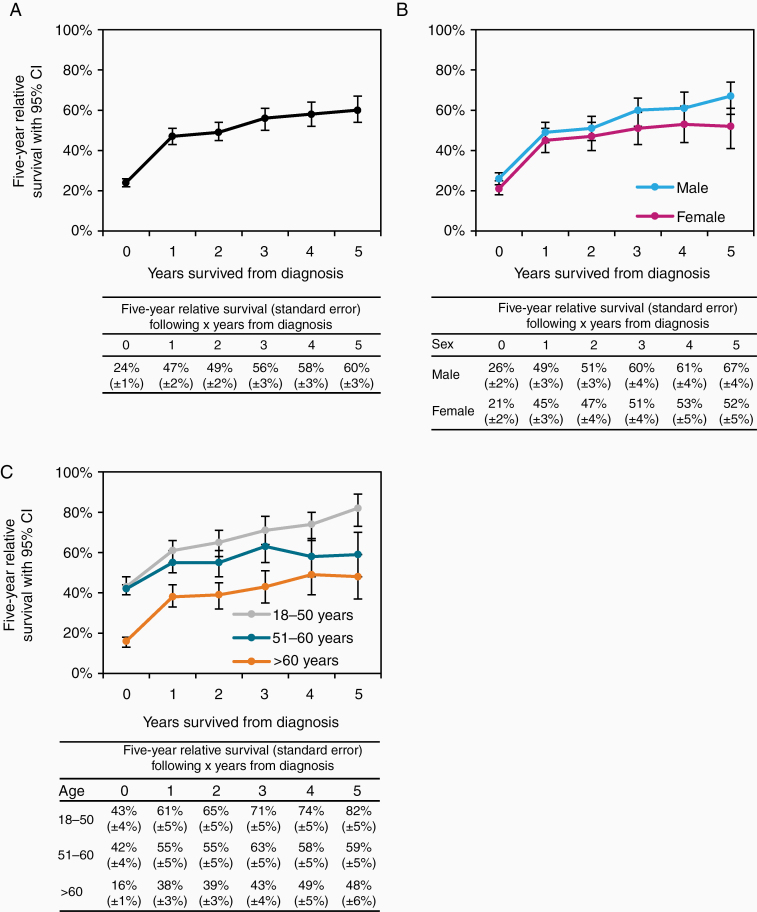
Conditional 5-year relative survival at each additional year survived after diagnosis among patients with primary central nervous system lymphoma in the Netherlands according to the overall cohort (A) and by sex (B) and age at diagnosis (C). The error bars for the point estimates of 5-year relative survival indicate 95% CIs. The table presented below each panel shows the point estimates of 5-year relative survival, with standard errors, according to the years survived after diagnosis. Under the hybrid approach, which incorporates elements of both the classical cohort and period approach, the survival experience of patients diagnosed during 1989–2018 who were at risk during the follow-up interval 1998–2019 was considered. More specifically, the classical cohort-based 1-year survival probability of patients diagnosed during 1998–2018 was combined with the period-based survival probabilities for the second to fifth years of follow-up among patients diagnosed during 1989–2018 who were alive at some point during the interval 1999–2019. As a result, 329 patients were excluded who died before the follow-up interval, leaving 1,658 of 1,987 patients who contributed to the survival estimates under the hybrid approach. As such, 5-year relative survival at diagnosis and the conditional 5-year relative survival at 1, 2, 3, 4, and 5 years postdiagnosis was considered through the survival experience of patients diagnosed during 1994–2018, 1993–2018, 1992–2017, 1991–2016, 1990–2015, and 1989–2014, respectively.

A total of 1,987 PCNSL patients were diagnosed in the Netherlands during 1989–2018 (53% males; median age 66 years; and 66% aged >60 years). Five-year relative survival increased sharply from 24% at diagnosis to 47% after 1 year postdiagnosis, and thereafter more gradually (60% at 5 years postdiagnosis; Figure 1A). This pattern was largely similar by sex, with no apparent sex-related differences in survival (Figure 1B). There were, however, clear age-related survival differences at diagnosis and for each additional year survived postdiagnosis (Figure 1C). Five-year relative survival at diagnosis was conspicuously lower for patients aged more than 60 years, as compared to patients aged 18–50 and 51–60 years. This age differential in survival largely persisted over time when considering the wideness of the 95% CIs. Patients aged 18–50 and 51–60 years had similar estimates of 5-year relative survival at diagnosis. However, significant differences in the conditional 5-year relative survival between these 2 age groups began to emerge at 5 years postdiagnosis.

To our knowledge, this is the first population-based study that examined up-to-date estimates of conditional 5-year relative survival among PCNSL patients. Age-specific information on conditional relative survival—along with information on specific risk factors—can provide PCNSL patients with relevant information regarding survivorship and support physicians to plan more tailored surveillance and follow-up activities. Moreover, this study provides clinically relevant insight into age-specific excess mortality at each additional year survived postdiagnosis that is both encouraging and daunting. Encouraging is the fact that excess mortality decreased over time at each additional year survived postdiagnosis, especially among patients aged 18–50 years at diagnosis. The results are daunting because conditional 5-year relative survival did not exceed 95%,^6^ indicating that PCNSL patients, regardless of age, continue to experience substantial excess mortality compared to equivalent groups from the general population. The continuing excess mortality might be explained by disease recurrence, late treatment-related sequelae, and the presence of comorbidity—especially in older patients. The main limitations of the study were the lack of detailed information throughout most of the registry to stratify survival estimates according to well-established prognostic indices and treatment.^1,4^ Also, the estimation of the conditional 5-year relative survival beyond 5-years postdiagnosis could not be reliably explored due to the comparatively small numbers of patients at risk after that time point. Nevertheless, this study highlights that future intervention studies in the upfront and salvage setting are needed to improve both the short- and long-term outcomes in PCNSL patients.
